# Supplementary material for: Genomic characterization and computational phenotyping of nitrogen-fixing bacteria isolated from Colombian sugarcane fields
Source: Sci Rep. 2021 Apr 28;11:9187. doi: 10.1038/s41598-021-88380-8 (PMC8080613; doi:10.1038/s41598-021-88380-8)
Supplement: Supplementary file 1 — Supplementary Information 1. [file 41598_2021_88380_MOESM1_ESM.pdf]

Supplementary Information For:

Genomic characterization and computational phenotyping of nitrogen-fixing bacteria isolated from Colombian sugarcane fields

Luz K. Medina-Cordoba, Aroon T. Chande, Lavanya Rishishwar, Leonard W. Mayer, Lina C. Valderrama-Aguirre, Augusto Valderrama-Aguirre, John Christian Gaby, Joel E. Kostka and I. King Jordan

Table of Contents

Supplementary Figure S1. Combinatoric platform for high-throughput enrichment of nitrogen fixing bacteria 2
Supplementary Figure S2. Phylogeny of the 16S rRNA genes for the bacterial isolates characterized here (SCK numbers) 3
Supplementary Figure S3. Computational phenotyping of previousl7yreported plant-associated *Klebsiella*. 4
Supplementary Table S1. Genome sequencing assembly and functional annotations. 5
Supplementary Table S2. Presence/absence calls for the three function-specific gene panels. N 6
Supplementary Table S3. Custom gene panels for plant growth promoting genes 6
Supplementary Table S4. List of genome sequence analysis software used in this manuscript. 7
Supplementary Table S5. Genome sequences for clinically-associated isolates from closely related *Klebsiella* species 8
Supplementary Table S6. Custom gene panel for nitrogen fixation genes. 8
Supplementary Materials and Methods 9
Sampling and cultivation of putative nitrogen-fixing bacteria from sugar cane. 9
Comparative genomic analysis. 10
Computational phenotyping. 11
Experimental validation 13
Acetylene reduction assay. 13
Phosphate solubilization assay. 14
Siderophore production assay. 14
Gibberellic acid production assay. 15
Indole acetic acid production assay. 15
Supplementary References 16

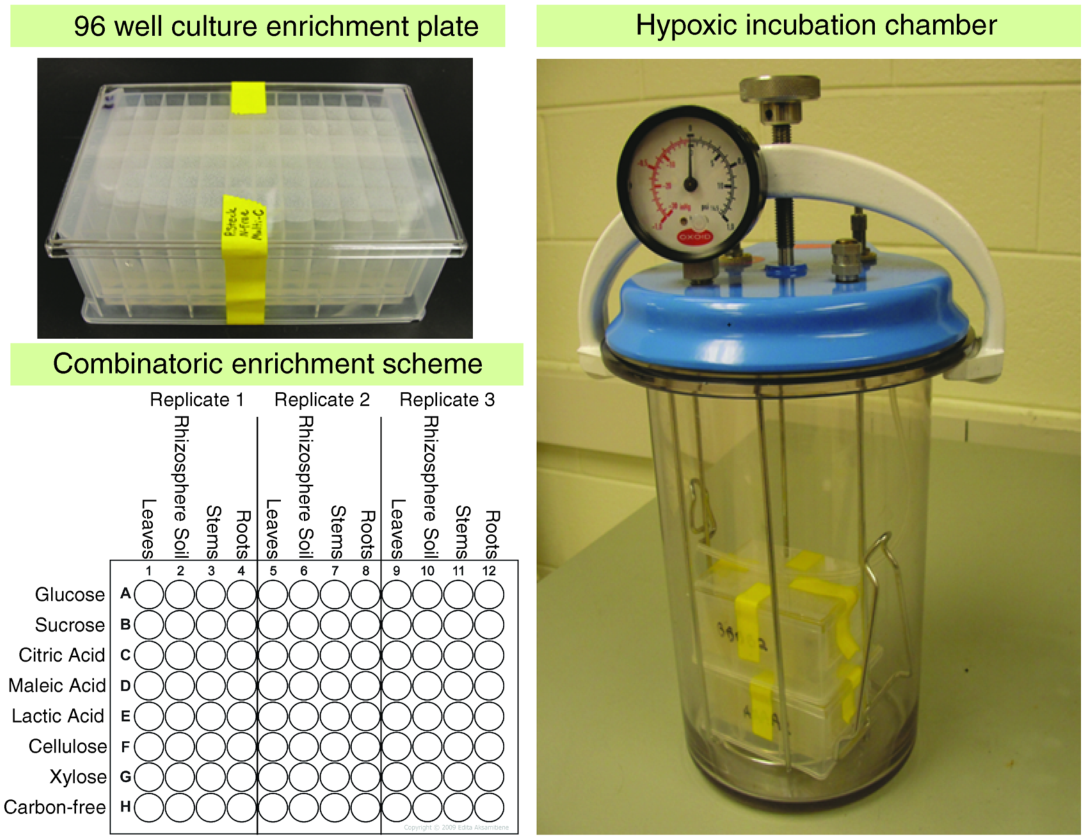

Supplementary Figure S1. **Combinatoric platform for high-throughput enrichment of nitrogen fixing bacteria.** A 96-well plate system was used to cultivate isolates from different sugarcane compartments (leaves, rhizosphere soil, stems, and roots) with nitrogen-free media based on multiple carbon sources (glucose, sucrose, citric acid etc.). Isolates were cultivated under atmospheric oxygen conditions and in a hypoxic atmosphere of 2% oxygen, using the incubation chamber shown.

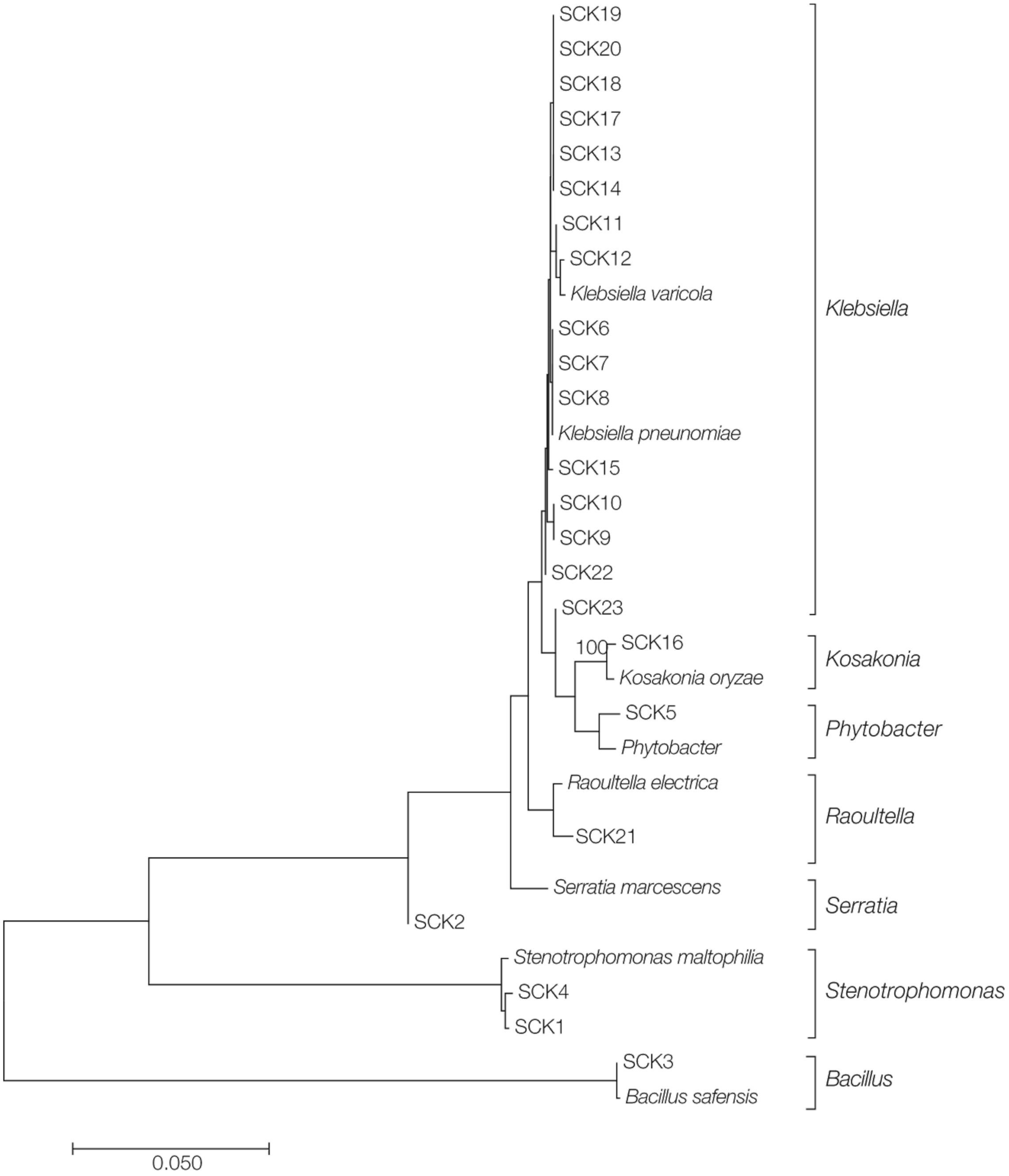

Supplementary Figure S2. **Phylogeny of the 16S rRNA genes for the bacterial isolates characterized here (SCK numbers) together with their most closely related bacterial type strains.** The phylogeny was reconstructed using p-distances between 16S rRNA sequences with the neighbor-joining method.

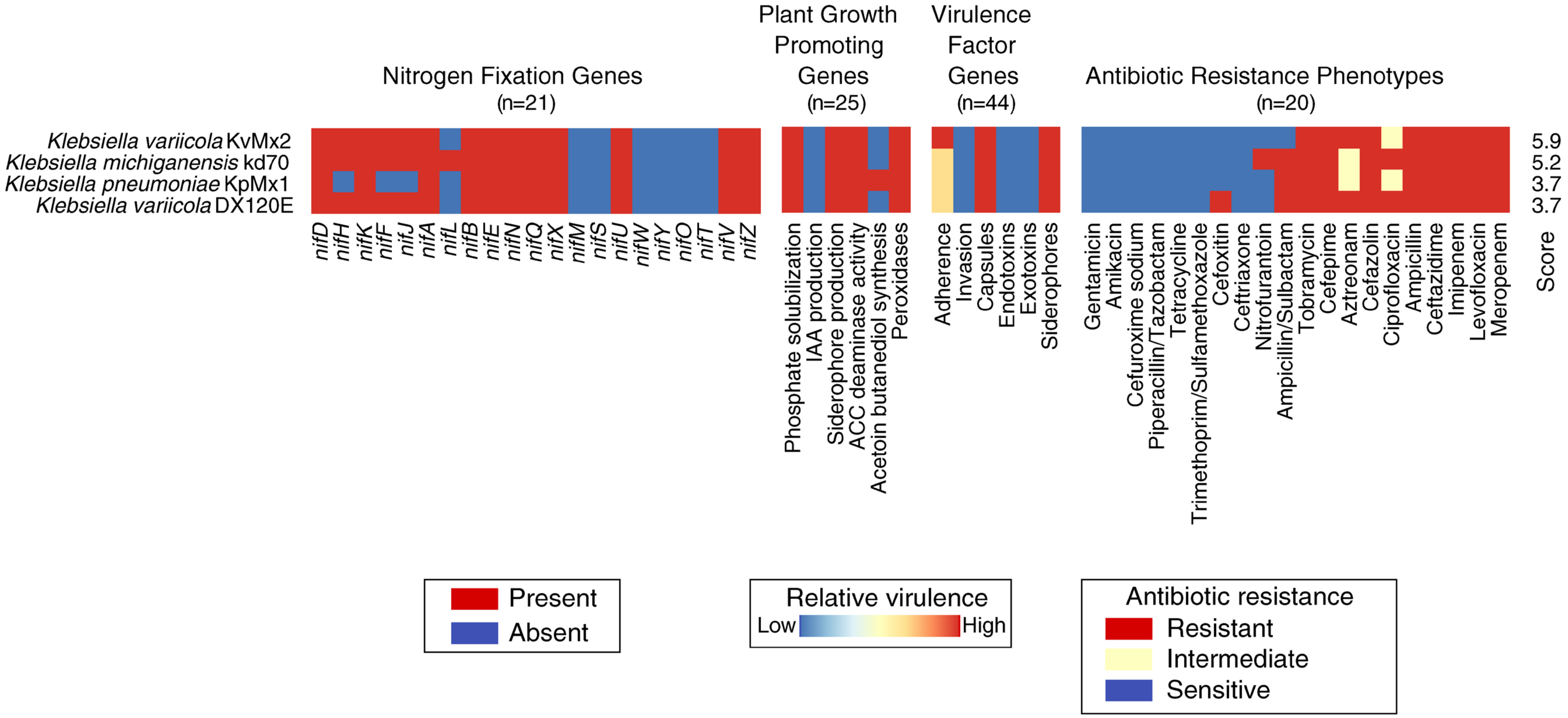

Supplementary Figure S3. **Computational phenotyping of previousl7yreported plant-associated *Klebsiella*.** The computational phenotyping approach used to evaluate our 22 environmental samples was employed to analyze four genome sequences of plant-associated *Klebsiella* that have been isolated and reported in previous studies.

Supplementary Table S1. **Genome sequencing assembly and functional annotations.** Isolate identifiers, NCBI BioSample identifiers, Genbank nucleotide accession numbers, and Genbank assembly accession numbers are shown for the 22 sugar cane-associated bacterial isolates characterized here.

Isolate ID	BioSample	GenBank accession	Assembly
**SCK1**	SAMN08687486	PYTV00000000	GCA_003028475.1
**SCK2**	SAMN08687489	PYTW00000000	GCA_003028435.1
**SCK3**	SAMN08687533	PYUA00000000	GCA_003028545.1
**SCK4**	SAMN08687959	PYTX00000000	GCA_003028535.1
**SCK5**	SAMN08687985	PYTY00000000	GCA_003028515.1
**SCK6**	SAMN08025861	PIOG00000000	GCA_002810545.1
**SCK7**	SAMN08025823	PIOH00000000	GCA_002810475.1
**SCK8**	SAMN08025819	PIOI00000000	GCA_002810535.1
**SCK9**	SAMN08025816	PIOJ00000000	GCA_002810495.1
**SCK10**	SAMN08025815	PIOK00000000	GCA_002810515.1
**SCK11**	SAMN08025808	PIOL00000000	GCA_002810575.1
**SCK12**	SAMN08025807	PIOM00000000	GCA_002810595.1
**SCK13**	SAMN08025806	PION00000000	GCA_002810615.1
**SCK14**	SAMN08025792	PIOO00000000	GCA_002810635.1
**SCK15**	SAMN08025791	PIBL00000000	GCA_002806645.1
**SCK16**	SAMN08025790	PYTZ00000000	GCA_003028445.1
**SCK17**	SAMN08025346	PIBM00000000	GCA_002806695.1
**SCK18**	SAMN08025338	PJDI00000000	GCA_002837655.1
**SCK19**	SAMN08025337	PIBN00000000	GCA_002806655.1
**SCK20**	SAMN08115734	PJDJ00000000	GCA_002837625.1
**SCK21**	SAMN08025336	PIBO00000000	GCA_002806725.1
**SCK22**	SAMN08115735	PJDK00000000	GCA_002837615.1

Supplementary Table S2. **Presence/absence calls for the three function-specific gene panels.** Nitrogen fixation, plant growth promoting, and virulence factors genes and predicted antibiotic resistance profiles are shown for the 22 sugar cane-associated bacterial isolates characterized here. These are the data that are visualized as a heatmap in Fig. 4. S = Predicted sensitive to antibiotic. I = Predicted intermediate sensitivy/resistance to antibiotic. R = Predicted resistant to antibiotic. **See file Medinaetal-ComputationalPhenotyping-Table S2.xlsx**

Supplementary Table S3. **Custom gene panels for plant growth promoting genes.** The 25 plant growth promoting genes used for computational phenotyping are shown along with brief descriptions of their function and their associated Genbank protein family accession numbers. The 25 genes are organized into six functional subcategories as shown. **See file Medinaetal-ComputationalPhenotyping-Table S3.xlsx**

Supplementary Table S4. **List of genome sequence analysis software used in this manuscript.**

Software	Version	URL	Function
Barrnap[1]	0.9	https://github.com/tseemann/barrnap	16S rRNA prediction
BCFtools[2]	1.7	http://www.htslib.org/	Calling SNPs and forming consensus sequences from alignment
BLAST[3]	2.2.28	https://blast.ncbi.nlm.nih.gov/Blast.cgi	Sequence database searches and alignment
BWA[4]	0.7.15	http://bio-bwa.sourceforge.net/	Read mapping
CGView[5]	1.0	http://wishart.biology.ualberta.ca/cgview/	Bacterial genome visualization and browsing
FastQC[6]	0.11.5	https://www.bioinformatics.babraham.ac.uk/	FASTQ quality control
MUSCLE[7]	3.8.425	https://www.drive5.com/muscle/	Multiple Sequence Alignment
Genome Workbench	2.12.0	https://www.ncbi.nlm.nih.gov/tools/gbench/	Compering and analyzing genetic data
MEGA[8]	7.1	https://www.megasoftware.net	DNA analysis and protein sequence data from species and populations
PATRIC3/mic_prediction	0b5494a	https://github.com/PATRIC3/mic_prediction	Minimum inhibitor concentration prediction for *Klebsiella* isolates
Mummer[9]	3.23	http://mummer.sourceforge.net/	DNA alignment and comparison
PATRIC[10]	3.5.20	https://www.patricbrc.org/	Genome and sequence analysis database
RAST[11]	N/A	http://rast.nmpdr.org/	Rapid annotation of microbial genomes
Samtools[2]	1.7	http://www.htslib.org/	Manipulating NGS alignments
SPAdes[12]	3.6	http://cab.spbu.ru/software/spades/	*de novo* assembly of the 22 isolate genomes
TaxaDiva[13]	0.11.3	https://github.com/lavanyarishishwar/taxadiva	Analysis of *nifH* amplicon sequences
Trimmomatic[14]	0.35	http://www.usadellab.org/cms/?page=trimmomatic	Remove low quality reads/regions
VSEARCH[15]	2.7.1	https://github.com/torognes/vsearch	Clustering and searching nucleotide sequences

Supplementary Table S5. **Genome sequences for clinically-associated isolates from closely related *Klebsiella* species.** Isolate identifiers are Genbank accession numbers are shown for the 29 clinically-associated *Klebsiella* isolates used for virulence profiling. See file Medinaetal-ComputationalPhenotyping-Table S5.xlsx

Supplementary Table S6. **Custom gene panel for nitrogen fixation genes.** The 21 bacterial nitrogen fixation genes (*nif*) used for computational phenotyping are shown along with brief descriptions of their function and their associated TIGRFAM protein family accession numbers (taken from the RAST annotation system). See file Medinaetal-ComputationalPhenotyping-Table S6.xlsx

Supplementary Materials and Methods

Sampling and cultivation of putative nitrogen-fixing bacteria from sugar cane. A 96-well plate system was used with modified Jensen's nitrogen-free media (Dipotassium phosphate 1000 mg/L, Magnesium sulphate 500 mg/L, Sodium chloride 500 mg/L, Ferrous sulphate 100 mg/L, Sodium molybdate 5 mg/L, and Calcium carbonate 2000 mg/L, pH 6.8), a selective media that allows for the enrichment of putative nitrogen-fixing bacteria that can grow in absence of nitrogen. 5 g/L of one of the following carbon sources were added to the modified Jensen's media prior to sterilization: cellobiose, sucrose, D-xlyose, arabinose, citrate, glucose, pectin, and malic acid. The 96-well plate system (Nunc® 96 DeepWell™ plate Volume 2.0 mL, polypropylene) was implemented with a combinatorial approach to the formulation and deposition of selective media such that multiple carbon sources (glucose, sucrose, citric acid, maleic acid, lactate, cellulose, and xylose) and inoculum types (leaves, rhizosphere soil, stem, and roots) were added to the nitrogen-free media to enrich for a variety nitrogen fixers from distinct plant-associated communities, which may grow under different conditions. Homogenized soil or tissue was diluted in phosphate buffered saline (pH 7) before inoculation into the media. Enrichment plates were incubated under normal atmospheric conditions, or in a hypoxic atmosphere of 2% oxygen, a condition which lowers cellular oxygen and allows the oxygen sensitive nitrogenase enzyme to function.

Droplets from individual wells of this high-throughput, combinatoric enrichment system were streaked for isolation with an inoculating loop onto petri plates made of the same nitrogen-free media, and the same carbon sources, in order to isolate individual colonies derived from a single cell of bacteria. The isolation plates were incubated under normal or hypoxic atmospheric conditions. Plates of putative nitrogen-fixing bacteria were visually inspected to search for colonies that showed distinct morphologies and were thereby more likely to represent unique bacterial strains. Colonies with unique morphologies were selected and screened for their nitrogen fixation potential by PCR amplification and sequencing of nitrogenase genes, *nifH*, a commonly used molecular marker for nitrogen fixation, using previously described methods [13]. Once a set of putative nitrogen-fixing isolates was obtained, then Ribosomal Intergenic Spacer Analysis (RISA)

[16] was used to identify genetically unique strains by PCR amplification with the primers S-D-Bact-1522-b-S-20 (5'-TGCGGCTGGATCCCCTCCTT-3') and L-D-Bact-132-a-A-18 (5'-CCGGGTTTCCCCATTCGG-3') and subsequent pattern visualization by agarose gel electrophoresis.

Comparative genomic analysis. Average nucleotide identity (ANI) values were calculated using the program MuMmer3 (v.3.23) [9], with the standard settings of DNA fragments showing >75% identity over >75% of their length used for ANI calculations. ANI cutoff values of 97% for species assignment and 85% for genus assignment were used. Isolate genome sequences were initially compared to a collection of ~7,000 NCBI RefSeq complete bacterial genome sequences for taxonomic assignment [17]. Isolates with >97% ANI to a single reference genome were assigned the same taxonomy. Isolates with >95% ANI to multiple reference genomes from the same genus were subject to an additional round of majority-rule based taxonomic assignment. For this procedure: 1) all completed or scaffold assemblies available for each genus was retrieved from NCBI Genbank, 2) ANIs for ambiguous isolates were computed against the corresponding genus-specific reference sets, and 3) majority-rule taxonomic assignments were made for the ambiguous isolates based on the most commonly observed species showing ANI values >95% for each genus. Pairwise ANI values for the bacterial isolates characterized here and their most closely related reference strains were converted to p-distances and used to reconstruct a neighbor-joining phylogenetic tree [18] using the program Molecular Evolutionary Genetics Analysis (MEGA, v.7.1) [8].

In support of ANI analysis, taxonomic assignment was also conducted by targeting small subunit ribosomal RNA (SSU rRNA) gene sequences. SSU rRNA sequences were extracted from the isolate genome sequences using the program Barrnap (v.0.9) [1]. For isolates where only partial or no 16S rRNA sequences were able to be extracted with Barrnap, full length 16S rRNA sequences were reconstructed by read mapping to the most closely related reference genome using BWA (v.0.7.15-r1140) [4] followed by consensus 16S rRNA sequence calling using SAMtools (v.1.7) and BCFtools (v.1.7) [2]. When multiple, full-length 16S rRNA sequences were present in a single isolate genome

sequence, VSEARCH (v.2.7.1) [15] was used to cluster the sequences and pick a single representative sequence centroid. The resulting full-length 16S rRNA sequences, along with 16S rRNA reference sequences from the NCBI 16S rRNA RefSeq database [17], were aligned using MUSCLE (v.3.8.425) [7] and a neighbor-joining phylogenetic tree [18], based on pairwise p-distances, was constructed using MEGA.

Nitrogenase enzyme encoding *nifH* gene sequences were extracted from isolate genome sequences, clustered, and taxonomically assigned using the TaxaDiva (v.0.11.3) method developed by our group [13]. For this approach, isolate genome sequence reads in FASTQ format were subjected to quality control and trimming followed by alignment to a *nifH* reference sequence database to produce *nifH* sequences and taxonomic assignments for each isolate. Details of this approach and the specific parameters used for genome-based *nifH* gene reconstruction and taxonomic assignment were previously described [13].

Whole genome sequence comparisons between bacterial isolates characterized here and the *K. variicola* type strain 342 were performed using BLAST+ (v.2.2.28) [3] and visualized with the program CGView (v.1.0) [19]. Isolate contig sequences were searched against the *K. variicola* 342 genome sequence, and BLAST best hits with >70% nucleotide identity were retained. The resulting BLAST best hits were parsed using a custom Perl script and mapped along the *K. variicola* 342 genome sequence.

Computational phenotyping. Gene panels were manually curated by searching the literature (NCBI PubMed) for genes implicated in nitrogen fixation and plant growth promotion. For nitrogen fixing (NF), all *n*=21 known bacterial nitrogen fixation (*nif*) genes were collected from the literature and gene (protein) annotation databases (NCBI and RAST, Table S6) [20,21]. For plant growth promotion (PGP), a total of *n*=25 genes from six distinct functional subcategories were collected in the same way. The resulting custom gene panels are shown in Table S2 (NF genes) and Table S3 (PGP genes). A gene panel containing subset of *n*=44 virulence factor (VF) genes that can be found among any of the bacterial isolates characterized here were taken from the Virulence Factors Database (VFDB) [22]. All of the gene (nucleotide) sequences in VFDB were compared

against the bacterial isolate genome sequences characterized here using BLAST [3], and VF genes that showed any hits >75% sequence identity over 75% of their length were retained for the gene panel. Gene (nucleotide) sequences from all three gene panels were used as queries to search against the bacterial isolate genome sequence assemblies using BLAST+, requiring the same criteria of >75% sequence identity over >75% of their length for a gene to be considered present in a genome. The presence of multiple gene copies was considered here, with each unique locus counting towards an isolate's score. Finally, VF genes were organized into operons to control for the genomic complexity of some VF phenotypes. For example, the siderophore aerobacterin is synthesized by 4 genes [23] which results in production of a single siderophore – when all four genes are present and co-located, a +1 is added to the Siderophore production phenotype score. Similarly, exotoxin is encoded by a single gene and the presence of this gene adds a +1 to the exotoxin score.

For antimicrobial resistance (AMR), the PATRIC3/mic prediction tool (commit: 0b5494a) [24] was used to predict minimum inhibitory concentrations (MIC) for *n*=20 antibiotic classes using the isolate genome sequences characterized here. Performance standards for antimicrobial susceptibility testing were taken from the Clinical & Laboratory Standards Institute (CLSI) [25] and used to convert the predicted MIC levels to sensitive, intermediate, and resistant phenotypes.

A composite score was developed to characterize each bacterial isolate genome sequence with respect to the presence/absence of genes from the NF, PGP, and VF gene panels along with the predicted AMR levels. For each bacterial isolate *i*, $Score_i = S.NF_i + S.PGP_i - S.VF_i - S.AMR_i$. $S.NF_i = \sum_{j=1}^{21} P_j$, where $P_j \in \{0,1\}$ corresponding to absence and presence of individual *nif* genes based on the results of BLAST+ searches against bacterial isolate genome assemblies. $S.PGP_i = \sum_{j=1}^{6} \frac{\sum_{k=1}^{k} count_k \times w_k}{\max(count_{j,i_{1\ldots22}})}$, where $count_k$ is the number of times any given PGP gene occurs in the bacterial isolate i , $j \in \left\{ \begin{matrix} Phospate\ solubilization, IAA\ production, Siderophore\ production, ACC\ deaminase\ activity, \\ Acetoin\ butanediol\ synthesis, Peroxiade \end{matrix} \right\}$,w_j is a category-specific weight, and $\max(count_{j,i_{1\ldots22}})$ is the maximum observed count of genes for each category of genes j across all 22 genomes. $S.VF_i =$

$\sum_{j=1}^{6} \frac{\sum_{k=1}^{k} count_k \times w_j}{\max(count_{j,i_{1\ldots22}})}$, where $count_k$ is the number of times any given VF gene or operon occurs in the bacterial isolate genome from category j, where $j \in \{Adherence, Invasion, Capsules, Endotoxins, Exotoxins, Siderophores\}$, w_k is a category-specific weight and $\max(count_{j,i_{1\ldots22}})$ is the maximum observed count of genes for each category of genes j across all 22 genomes. Category-specific weights for the PGP and VF gene panels are $w_i = 1$ for all genes except siderophore genes, which are present in both the PGP and VF panels. Their relative contributions to each phenotype were approximated to yield gene-panel specific weights. PGP siderophore genes are weighted as $w = 0.7$, and VF siderophore genes are weighted as $w = 0.3$, in light of their known relative contributions to plant growth promotion versus virulence. $S.AMR_i = \sum_{j=1}^{20} A_j$, where $A_j \in \{0, 0.5, 1\}$, corresponding to predicted sensitive, intermediate, and resistant AMR phenotypes. Each of the four sets of functional category-specific scores were normalized by the maximum score such that each category contributed equally to the final genome score.

An additional set of VF genome scores, computed using the same gene panel and formula described above, were compared for the environmental bacterial isolates characterized here together with a set of genome sequences from 29 clinically-associated isolates from closely related *Klebsiella* species taken from the NCBI RefSeq database (Table S5).

Experimental validation

Acetylene reduction assay. Potential nitrogen fixation activity was quantified using the acetylene reduction assay [26]. Strains were cultivated in pressure tubes containing 10 ml of nitrogen-free Jensen's broth and sealed with a rubber stopper. Cultures were incubated for 120 hours at room temperature in a shaker (at 1800 rpm), and growth was monitored by optical density at 600 nm wavelength. Acetylene was added to the headspace (2.5 ml of acetylene was added to a 25 ml headspace), and 100 µl of headspace was sampled for quantification of ethylene production via gas chromatography after 120 hours. Rates

(nmol C_2H_4/ml/h) were determined by linear regression of ethylene concentrations over time after comparison to a standard curve. Controls: *Azotobacter vinelandii* (positive) and *Escherichia coli* (negative).

Phosphate solubilization assay. Plant growth promotion phenotypes were verified using a series of cultivation-based assays. Phosphate solubilization activity was evaluated using Pikovskaya's agar medium as previously described [27]. Isolate cultures from LB agar medium were resuspended in LB medium and diluted to an OD600 of 0.5, which corresponds to an approximate cell density of 4×10^8 cells/ml. 100 µl of this suspension was pelleted at 15,000 *g* at room temperature and buffer exchanged into 5 ml of 0.1% Peptone Salt solution (pH 8). 50 µl of the 0.1% Peptone Salt cell suspension was inoculated onto a sterile filter paper disk. Inoculated filters were cultivated on Pikovskaya's agar medium for 48 hours. After 48 hours of growth, the potential to solubilize phosphate was determined by the formation of a clear zone around each colony. A phosphate solubilization index was calculated by measuring the colony and halo zone using the following formula: $(Colony\ diameter + Halo\ zone\ diameter) / (Colony\ diameter)$. According to [28], phosphate solubilization efficacy can be approximated with values of 1 to 2 considered as low solubilizers, values from 2 to 3 considered as medium solubilizers, and values above 3 considered as high solubilizers. Controls: *Pseudomonas aeruginosa* ATCC 2785 (positive) and *Escherichia coli* (negative)

Siderophore production assay. Siderophore production was assessed using the chrome azurol S (CAS) media as previously described [29,30]. CAS is a blue indicator dye sensitive to the oxidation state of iron and iron (III) and LB media. Isolates were cultivated on CAS media by adding 50 µl of each suspension (same culture density and media used for inoculum for Phosphate solubilization assay) of isolates onto a sterile paper disc. Isolates exhibiting a yellowish-orange halo after 5 days of incubation at 28 ± 2 °C were considered positive for siderophore production. A siderophore production index was calculated by measuring the colony and halo diameter using the following formula ($Colony$

$diameter + Halo\ zone\ diameter) / (Colony\ diameter)$. Controls: *Escherichia coli* ATCC 35218 (positive) and *Bacillus safensis* (negative).

Gibberellic acid production assay. Gibberellic acid (GA) production was assessed using the enzyme-linked immunosorbent assay kit MyBiosource (MBS2000244) for Gibberellic acid following the manufacturer's recommended protocol [31]. The concentration of the Gibberellic acid standard in the stock solution is 10,000ng/mL. The assay utilizes a competitive inhibition reaction between biotin labeled gibberellic acid and unlabeled gibberellic acid (standards or samples) with a pre-coated antibody specific to gibberellic acid. Briefly, GA production was calculated by averaging triplicate reading (optical density) for each standard, control, and samples using a standard curve for linear regression. After incubation the unbound conjugate is washed off and detection reagent added. Following incubation, the optical density at 450nm was read using a plate reader to quantify the amount of GA. Controls: *Azotobacter vinelandii* (positive) and *Stenotrophomonas sp* (negative).

Indole acetic acid production assay. Indole acetic acid (IAA) production was evaluated using a colorimetric method according to [32]. Each isolate was cultivated in tryptophan broth and incubated for 48 hours. After the incubation, 10 ml of culture was centrifuged, and 2 ml of the supernatant was combined with 2ml of Salkowski reagent (0.5M $FeCl_3$ and 70% perchloric acid). A standard curve was prepared of IAA (Sigma-Aldrich) and optical density was measured at 540 nm.

Supplementary References

1 barrnap: BAsic Rapid Ribosomal RNA Predictor v. 0.9 (2018).

2 Li, H. *et al.* The Sequence Alignment/Map format and SAMtools. *Bioinformatics* 25, 2078-2079, doi:10.1093/bioinformatics/btp352 (2009).

3 Camacho, C. *et al.* BLAST+: architecture and applications. *BMC Bioinformatics* 10, 421, doi:10.1186/1471-2105-10-421 (2009).

4 Li, H. & Durbin, R. Fast and accurate short read alignment with Burrows-Wheeler transform. *Bioinformatics* 25, 1754-1760, doi:10.1093/bioinformatics/btp324 (2009).

5 Stothard, P. & Wishart, D. S. Circular genome visualization and exploration using CGView. *Bioinformatics* 21, 537-539, doi:10.1093/bioinformatics/bti054 (2005).

6 Andrews, S. *FastQC a quality control tool for high throughput sequence data*, <http://www.bioinformatics.babraham.ac.uk/projects/fastqc/> (

7 Edgar, R. C. MUSCLE: a multiple sequence alignment method with reduced time and space complexity. *BMC Bioinformatics* 5, 113, doi:10.1186/1471-2105-5-113 (2004).

8 Kumar, S., Stecher, G., Li, M., Knyaz, C. & Tamura, K. MEGA X: Molecular Evolutionary Genetics Analysis across computing platforms. *Mol Biol Evol* 35, 1547-1549, doi:10.1093/molbev/msy096 (2018).

9 Kurtz, S. *et al.* Versatile and open software for comparing large genomes. *Genome Biol* 5, R12, doi:10.1186/gb-2004-5-2-r12 (2004).

10 Wattam, A. R. *et al.* PATRIC, the bacterial bioinformatics database and analysis resource. *Nucleic Acids Res* 42, D581-591, doi:10.1093/nar/gkt1099 (2014).

11 Aziz, R. K. *et al.* The RAST Server: rapid annotations using subsystems technology. *BMC Genomics* 9, 75, doi:10.1186/1471-2164-9-75 (2008).

12 Bankevich, A. *et al.* SPAdes: a new genome assembly algorithm and its applications to single-cell sequencing. *J Comput Biol* 19, 455-477, doi:10.1089/cmb.2012.0021 (2012).

13 Gaby, J. C. *et al.* Diazotroph Community Characterization via a High-Throughput nifH Amplicon Sequencing and Analysis Pipeline. *Appl Environ Microbiol* 84, doi:10.1128/AEM.01512-17 (2018).

14 Bolger, A. M., Lohse, M. & Usadel, B. Trimmomatic: a flexible trimmer for Illumina sequence data. *Bioinformatics* 30, 2114-2120 (2014).

15 Rognes, T., Flouri, T., Nichols, B., Quince, C. & Mahe, F. VSEARCH: a versatile open source tool for metagenomics. *PeerJ* 4, e2584, doi:10.7717/peerj.2584 (2016).

16 Cardinale, M. *et al.* Comparison of different primer sets for use in automated ribosomal intergenic spacer analysis of complex bacterial communities. *Appl Environ Microb* 70, 6147-6156, doi:10.1128/Aem.70.10.6147-6156.2004 (2004).

17 O'Leary, N. A. *et al.* Reference sequence (RefSeq) database at NCBI: current status, taxonomic expansion, and functional annotation. *Nucleic Acids Res* 44, D733-745, doi:10.1093/nar/gkv1189 (2016).

18 Saitou, N. & Nei, M. The neighbor-joining method: a new method for reconstructing phylogenetic trees. *Mol Biol Evol* 4, 406-425, doi:10.1093/oxfordjournals.molbev.a040454 (1987).

19 Grant, J. R., Arantes, A. S. & Stothard, P. Comparing thousands of circular genomes using the CGView comparison tool. *BMC Genomics* 13, 202, doi:10.1186/1471-2164-13-202 (2012).

20 Aziz, R. K. *et al.* The RAST Server: rapid annotations using subsystems technology. *BMC genomics* 9, 75 (2008).

21 Benson, D. A. *et al.* GenBank. *Nucleic Acids Res* 43, D30-35, doi:10.1093/nar/gku1216 (2015).

22 Chen, L., Zheng, D., Liu, B., Yang, J. & Jin, Q. Hierarchical and refined dataset for big data analysis--10 years on. *Nucleic Acids Res* 44, D694-697. (2016).

23 Bailey, D. C. *et al.* Structural and functional delineation of aerobactin biosynthesis in hypervirulent *Klebsiella pneumoniae*. *J Biol Chem* 293, 7841-7852 (2018).

24 Nguyen, M. *et al.* Developing an in silico minimum inhibitory concentration panel test for *Klebsiella pneumoniae*. *Sci Rep* 8, 421, doi:10.1038/s41598-017-18972-w (2018).

25 Wayne, P. A. *CLSI. Performance Standards for Antimicrobial Susceptibility Testing; Twenty-Fifth Informational Supplement*. (2015).

26 Rice, W. A. & Paul, E. A. The acetylene reduction assay for measuring nitrogen fixation in waterlogged soil. *Can J Microbiol* 17, 1049-1056 (1971).

27 Chakdar, H., Dastager, S. G., Khire, J. M., Rane, D. & Dharne, M. S. Characterization of mineral phosphate solubilizing and plant growth promoting bacteria from termite soil of arid region. *3 Biotech* 8, 463, doi:10.1007/s13205-018-1488-4 (2018).

28 Silva Filho, G. N. & Vidor, C. Solubilização de fostatos por microrganismos na presença de fontes de carbono. *Revista Brasileira de Ciência do Solo* 24, 311-319 (2000).

29 Schwyn, B. & Neilands, J. B. Universal chemical assay for the detection and determination of siderophores. *Anal Biochem* 160, 47-56 (1987).

30 Lakshmanan, V. *et al.* A natural rice rhizospheric bacterium abates arsenic accumulation in rice (*Oryza sativa* L.). *Planta* 242, 1037-1050 (2015).

31 de Salamone, I. E. G., Hynes, R. K. & Nelson, L. M. Cytokinin production by plant growth promoting rhizobacteria and selected mutants. *Can J Microbiol* 47, 404-411 (2001).

32 Gordon, S. A. & Weber, R. P. Colorimetric estimation of indoleacetic acid. *Plant Physiol* 26, 192-195 (1951).
